# The Accuracy of Hestia and Simplified PESI to Predict the Prognosis in Pulmonary Embolism: Systematic Review with Meta-analysis

**DOI:** 10.1055/a-1942-2526

**Published:** 2022-10-23

**Authors:** Miguel Palas, Beatriz Valente Silva, Cláudia Jorge, Ana G. Almeida, Fausto J. Pinto, Daniel Caldeira

**Affiliations:** 1 Faculdade de Medicina, Universidade de Lisboa, Lisboa, Portugal; 2 Centro Cardiovascular da Universidade de Lisboa (CCUL@RISE), CAML, Faculdade de Medicina, Universidade de Lisboa, Lisboa, Portugal; 3 Serviço de Cardiologia, Hospital Universitário de Santa Maria (CHULN), Lisboa, Portugal; 4 Laboratory of Clinical Pharmacology and Therapeutics, Faculdade de Medicina, Universidade de Lisboa, Lisboa, Portugal

**Keywords:** low-risk pulmonary embolism, outpatient treatment, mortality, Hestia, sPESI

## Abstract

**Introduction** Pulmonary embolism (PE) patients at low risk of early complications may be considered for early discharge or home treatment. Last decades evidence has been growing about the safety of several clinical prediction rules for selecting those patients, such as simplified Pulmonary Embolism Severity Index (sPESI) and Hestia Criteria. The aim of this review was to compare the safety of both strategies regarding 30-days mortality, venous thromboembolism recurrence and major bleeding.

**Methods** A systematic literature search was conducted using MEDLINE, CENTRAL and Web of Science on 6 ^th^ January 2022. We searched for studies that applied both Hestia Criteria and sPESI to the same population. Sensitivity, specificity and diagnostic odds ratio were calculated for both stratification rules. Both Hestia and sPESI criteria of low risk were evaluated to set the number of patients that could be misclassified for each 1000 patients with PE. The estimates were reported with their 95% confidence intervals (95%CI).

**Results** This systematic review included 3 studies. Only mortality data was able to be pooled. Regarding mortality, the sensitivity, specificity and diagnostic odds ratio was 0.923 (95%CI: 0.843–0.964), 0.338 (95%CI: 0.262–0.423) and 6.120 (95%CI: 2.905–12.890) for Hestia Criteria; and 0.972 (95%CI: 0.917–0.991), 0.269 (95%CI: 0.209–0.338) and 12.738 (95%CI: 3.979–40.774) for sPESI score. The negative predictive values were higher than 0.977. The risk of misclassification of high-risk patients in low risk was 5 (95%CI: 3–11) with Hestia and 2 (95%CI: 1–6) with sPESI, for each 1000 patients with PE in terms of mortality.

**Conclusion** The risk of misclassification of patients presenting with low-risk pulmonary embolism with the intent of early discharge or home treatment with both Hestia Criteria and sPESI score is low and these data supports methods for this purpose.

## Introduction

Venous thromboembolism (VTE), clinically presenting as deep vein thrombosis or pulmonary embolism (PE), is the third most frequent acute cardiovascular syndrome, standing below myocardial infarction and stroke. 1 The annual incidence rates for PE range from 39 to 115 per 100.000 population, and a rising tendency is expected in the upcoming years. 2 3 Even though PE related mortality has decreased, it still represents a significant burden to people and healthcare systems globally. 1

Apart from hemodynamically unstable patients requiring specific fibrinolytic therapy, treatment of PE is mainly based on anticoagulation to avoid recurrence and promote the natural fibrinolysis. 1 Historically, hospitalization was considered appropriate in PE patients due to VTE recurrence and bleeding risks. However, in the last decade, evidence on the safety of outpatient treatment of selected low-risk patients with PE has been accumulating. International guidelines suggest early discharge and outpatient treatment for patients at low risk for short-term adverse outcomes. 1

Several clinical rules have been validated to identify low-risk patients who could be eligible for home treatment, allowing the early resume of patients to their everyday life and contributing to the reduction of health systems costs. Some of the most consensual stratification rules are the simplified Pulmonary Embolism Severity Index (sPESI) 4 and the Hestia criteria. 5

The simplified PESI (sPESI) score rose from the need to simplify the classical tool Pulmonary Embolism Severity Index (PESI) which evaluates 11 important clinical features each one with its own weight for risk stratification. 6 The sPESI evaluates only 6 features and assigns a score for each of the following: age > 80 years, history of cancer, chronic cardiopulmonary disease, systolic blood pressure < 100 mm Hg, heart rate ≥ 110 beats per minute, or oxygen saturation < 90%. 4 Patients with sPESI of 0 can be treated at home, providing the proper follow-up and anticoagulant therapy. 4 7

In the Hestia Criteria, triage for outpatients' treatment of PE was performed using an 11-point questionnaire considering aspects related to PE severity, risk of bleeding, comorbidities, and feasibility of home treatment. 1 5 Only patients without any of the criteria were deemed to be eligible for home-treatment. 1 5

The sPESI score was developed to identify patients at low risk of early mortality, while the Hestia criteria was tailored to patients for outpatient treatment. Thus, Hestia criteria consider social aspects that may be determinant in the clinical practice when considering the eligibility for home treatment, which are left out by sPESI. Otherwise, sPESI considers certain comorbidities, such as cancer, as exclusion criteria for low-risk pulmonary embolism, although it is not clear how treatment as inpatients may impact the outcomes in these patients. 8

Although there is some evidence about the safety of the Hestia rule and sPESI score on selecting patients for early discharge, a review that gathers all existing data are needed to provide more insured decision-making for clinicians. This systematic review aimed to compare the predictive value on early mortality of the Hestia Criteria and sPESI on patients presenting with low-risk pulmonary embolism.

## Methods

### Eligibility Criteria

Relevant studies were systematically reviewed, and extractable data were analyzed following the Preferred Reporting Items for Systematic Reviews and Meta-Analyses (PRISMA) guidelines. This systematic review considered longitudinal studies that applied both Hestia Criteria and sPESI score to the same population of patients presenting with PE. We did not compare these scores with others as these are recognizably the most practical and used tools for risk stratification aiming to identify patients for early discharge. The selected studies should have information that allowed the assessment of the predictive value of these stratification rules for 30-day mortality after the acute PE and, whenever possible, data on hemorrhage and venous thromboembolism recurrence.

### Search Methods and Data Collection

A database search was done on 6 ^th^ January 2022 through MEDLINE, Cochrane Central Register of Controlled Trials (CENTRAL) and Web of Science that matched the keywords relevant to the review (“Hestia” or “sPESI” or “simplified PESI), without any language or timeline restriction. A study selection was initially performed based on study abstracts. Articles that did not match the area of interest were excluded. Studies that only used one of the stratification rules (Hestia Criteria or sPESI score) were also excluded. Articles that could not be excluded based solely on its abstract were left for full-text assessment.

Two reviewers independently screened the titles and abstracts identified by the literature search. Full-text papers selected from the search results were also independently screened by two reviewers. Any disagreements were resolved by consensus. A search through reference lists of studies was made to find additional articles that could be included in the systematic review. From this search, there were no additional articles suitable for inclusion in the review.

**Fig. 1:**
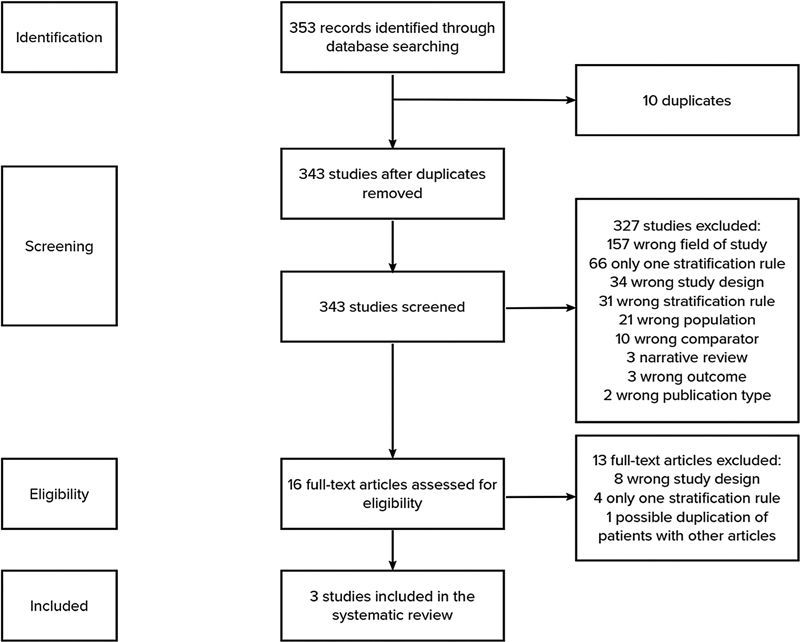
Flowchart outlining the protocol adopted in this systematic review.

The included studies collected information on study design, demographic data, prognostic rules evaluated and information on mortality rates at least 30 days after the acute event, and, whenever possible, data regarding bleeding-related outcomes and VTE recurrence. The study flowchart is represented in Fig. 1 .

### Data Extraction and Assessment of Methodological Quality

Two independent reviewers extracted the data from the articles and any disagreements were resolved by consensus.

Methodological quality of eligible studies was evaluated independently by two authors using the revised tool for quality assessment of diagnostic accuracy studies (QUADAS-2). QUADAS-2 is an updated version of the original QUADAS, including four aspects of patient selection, index test, reference standard, and flow and timing, which has a more accurate bias level and applicability to the original research than the original QUADAS.

### Statistical Analysis and Data Synthesis

This review aimed to assess the sensitivity, specificity, and negative predictive value of the Hestia Criteria and sPESI score on selecting patients presenting with low risk pulmonary embolism.

These criteria aim to detect patients at risk of early mortality to exclude those patients from an early discharge or home-treatment strategy. For statistical analysis, a true positive was defined as a patient classified into a high-risk category that dies within the first 30 days after presentation with PE. The primary outcome was early mortality. The incidence of major bleeding and VTE recurrence were analyzed as secondary outcomes.

For each study, we retrieved data and calculated the sensitivity and specificity of both stratification rules on the prediction of 30-days mortality after the diagnosis of PE. A weighted pooled analysis was performed and presented in forest plots and receiver operating characteristic plane. Diagnostic odds ratio, an overall diagnostic performance measure, was calculated for each study and pooled for a global estimate for each test in paired comparisons. This estimate combines both positive and negative likelihood ratios and shows the probability that patients with a positive test die within the first 30 days following the presentation of the PE, compared with those with a negative test. We expressed forest plot data with their central estimates and 95% confidence intervals (95% CI).

## Results

### Results of the Search and Included Studies

After excluding duplicates and a preliminary screening, 16 articles were selected for inclusion. Most of these studies were not readily excluded on a primary approach to retrieve data necessary to derive the patients' scores and mortality. From this set of articles, 13 were excluded due to the absence of information from every patient individually. Three studies were included in the systematic review: one has a retrospective design 9 , and two are prospective. 10 11 These studies were published between 2016 and 2022 and evaluated 1608 patients diagnosed with PE, with a weighted mean age of 71 years old. The studies' main characteristics are represented in Table 1 . One study (Vanni et al. 10 ) evaluated 30-day mortality and recurrence of symptomatic venous thromboembolism or major hemorrhage. The other two included studies only reported follow-up information regarding mortality. Additional characteristics and main results of the included studies are summarized in supplementary filles ( Supplementary Table S1 (online only)).

**Table 1:** Characteristics of included studies

Study	Study design	Patients	Primary outcome	Index tests	Anticoagulation allowed
Weeda et. al 9	Retrospective	573 in the 30-day mortality cohort; Mean age 64 years old	In-hospital and **30-day mortality**	**Hestia** , **sPESI** , PESI, IMPACT	Not mentioned
Vanni et. al 10	Prospective	547; 178 had early discharge upon decision of the attending physician; Mean age 76 years old	Recurrence of symptomatic venous thromboembolism, major hemorrhage, and **30-day mortality**	**Hestia** , **sPESI**	Unfractionated heparin, low molecular weight heparin, fondaparinux, warfarin and direct oral anticoagulants
Quezada et. al 11	Prospective	488; Mean age 74 years old	**30-day mortality**	**Hestia** , **sPESI** , PESI, Clinical Gestalt	Not mentioned

Abbreviations: IMPACT, In-hospital mortality for pulmonary embolism using claims data; PESI, pulmonary embolism severity index; sPESI, simplified pulmonary embolism severity index.

### Risk of Bias

The quality assessment results for the included studies (Quality Assessment of studies of Diagnostic Accuracy included in Systematic reviews – QUADAS-2) is summarized in Table 2 . The details on methodological assessment of the included studies are presented in the supplementary file (online only).

**Table 2:** Quality assessment by QUADAS-2 criteria

Author	Year	Risk of bias					Applicability			
		Patient Selection	Index tests		Reference Standard	Flow and Timing	Patient Selection	Index test		Reference Standard
			Hestia	sPESI				Hestia	sPESI	
Weeda et. al 9	2016	+	+	+	+	+	+	+	+	+
Vanni et. al 10	2018	+	+	+	+	+	+	+	+	+
Quezada et. al 11	2019	+	+	+	+	+	+	+	+	+

+, low-risk of bias/concern; -, high-risk of bias/concern

All included studies were considered low risk of bias with low concern regarding applicability. The only item worth mentioning occurs in Vanni et al. study, which included patients with an incidental diagnosis of pulmonary embolism. This fact may partially affect the risk of bias regarding patient' selection, but the overall risk in this category remains as low.

### Hestia Criteria and sPESI Score: Mortality

The sensitivity, specificity, false positive rate, diagnostic odds ratio and positive and negative likelihood ratio for Hestia Criteria and sPESI score is represented in supplementary file ( supplementary table S2 and S3 (online only)). The variation between studies for specificity and sensitivity for Hestia Criteria and sPESI score is represented in supplementary files ( supplementary figure S1 – S4 (online only)).

The Hestia Criteria sensitivity for 30-day mortality was 0.923 (95% CI 0.843–0.964). Its specificity for this same outcome was 0.338 (95% CI 0.262–0.423). The diagnostic odds ratio was 6.120 (95% CI 2.905- 12.890). Negative predictive value along the studies ranged from 97.7% (95% CI 93.0–99.4%) and 100% (95% CI 97.1–100%).

The sPESI score sensitivity for 30-day mortality was 0.972 (95% CI 0.917–0.991). Its specificity for this same outcome was 0.269 (95% CI 0.209–0.338). The diagnostic odds ratio was 12.738 (95% CI 3.979–40.774). Negative predictive value along the studies ranged from 99.0% (95% CI 93.4–99.9%) and 99.4% (95% CI 96.4–100%).

The positive predictive value ranges from 8.1 (5.7–11.3) to 9.6 (9.0–10.1) for sPESI, and from 7.9 (5.4–11.3) to 12.2 (10.8–13.6) for Hestia.

**Fig. 2:**
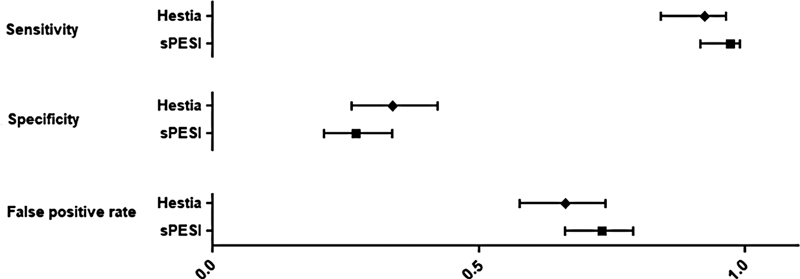
Hestia Criteria and sPESI score sensitivity, specificity and false-positive rate comparisons. sPESI: simplified Pulmonary Embolism Severity Index.

**Fig. 3:**
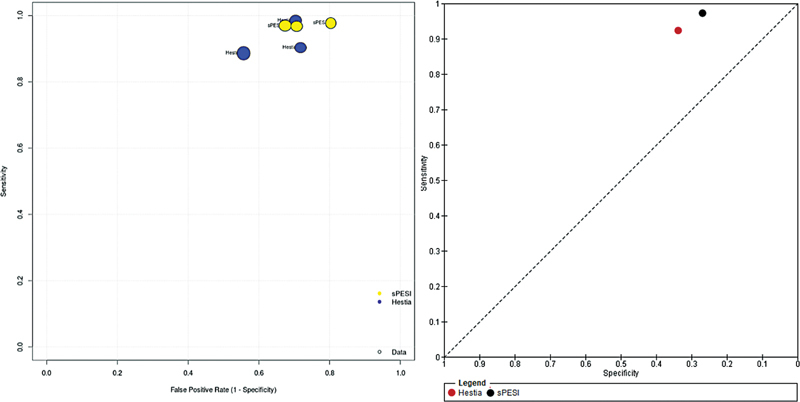
Estimates of individual studies (left) and pooled data (right) in Receiver-Operating Characteristic Curve, comparing Hestia Criteria and sPESI score. sPESI: simplified Pulmonary Embolism Severity Index.

**Fig. 4:**
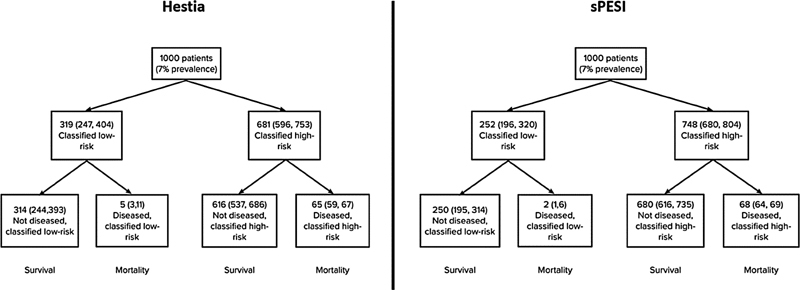
show the survival outcomes for Hestia Criteria and sPESI score according to the risk-stratification, assuming an average of 7% mortality. For every 1000 patients with PE, Hestia misclassifies 5 patients with low mortality risk, while sPESI misclassifies 2 patients.

Fig. 2 represents the comparison of Hestia Criteria and sPESI scores' sensitivity and specificity. Fig. 3 illustrates the estimates in the receiver-operating characteristic curve of Hestia Criteria and sPESI score. Specificity of Hestia Criteria and sPESI score are compared in supplementary files ( Supplementary figure S5 ). Hestia Criteria and sPESI score estimates in ROC plane is represented in supplementary files ( supplementary figure S6 (online only)). ( Fig. 4 )

### Major Bleeding and Venous Thromboembolism Recurrence

Only one of the included studies ( *Vanni et. al* 10 ) included major bleeding and VTE recurrence as primary outcomes. This study included 547 patients; of those, Hestia Criteria and sPESI score selected 228 (41.7%; 95% CI 37.5–46.0%) and 100 patients (18.3%; 95% CI 15.1–21.8%) to early discharge, respectively.

In patients classified as low risk by Hestia Criteria, one patient (0.4%; 95% CI 0.01–2.4%) had recurrent VTE, with a non-fatal deep vein thrombosis diagnosis. Of note that this patient had reduced the dose of the prescribed low-molecular-weight heparin by himself. No cases of major bleeding were reported.

In patients classified as low risk by sPESI score, two patients (2.0%; 95% CI 0.2–7.0%) had recurrent VTE. Likewise, none of the patients had major bleeding during the 30-day follow-up.

## Discussion

This systematic review aimed to compare the predictive value of Hestia Criteria and sPESI score to correctly exclude patients at high risk of early mortality in patients presenting with low-risk acute PE according to each of these tools. The clinical relevance of this study is to assess the accuracy of these risk-stratification rules to select patients for home treatment safely. Although none of the included studies is powered to answer this question, as none of them selected patients to early discharge based on sPESI score or Hestia Criteria, this review may give an insight on the outcomes that this strategy may comprise.

Overall, the sPESI score showed a higher sensitivity for early-mortality prediction in low-risk PE patients than Hestia Criteria, although this fails to reach statistical significance. Thus, sPESI score tends to be better in the selection of low-risk PE patients that will survive. However, the Hestia Criteria demonstrated a similarly high sensitivity. The high sensitivity of both strategies translates into high negative predictive values, meaning that if any of these sets of criteria select a patient for a low-risk category, there is a high probability of survival. Regarding specificity, even though the Hestia Criteria had better performance, both prognostic rules showed low specificity, as they both select patients for a high-risk category that turn out to survive.

From the clinician perspective, the high negative predictive value may be the most important test characteristic, allowing a safe early discharge. Even though these criteria rule out many patients who would be candidates for home-treatment, they selected low-risk patients with a high probability of survival 30-days after acute PE.

The HOME-PE was a randomized open-label non-inferiority trial that compared the Hestia Criteria and the sPESI score as triage strategies for selecting patients for home treatment. 12 This study highlights that about one-third of low-risk patients with PE can be safely treated at home and demonstrated the non-inferiority of the Hestia Criteria compared with the sPESI score regarding all-cause death, 30-day rate of recurrent VTE or major bleeding. In this study, a greater proportion of patients were eligible for home treatment using the sPESI score compared with the Hestia Criteria (48.4% and 39.4%, respectively) – however, due to the physicians' possibility to overrule the triaging tool, the final proportions of patients who discharged within 24 hours were similar with both strategies (38.4% in the Hestia Criteria group and 36.6% in the sPESI score group; *p* = 0.42). 12 The higher prevalence of overruling (in favor of hospitalization) in the arm of sPESI score strategy comparing to the Hestia Criteria (28.5% vs 3.4%) was mainly related to concomitant illness and social reasons. This fact highlights the importance of considering additional medical and social conditions to decide home treatment, and it may suggest that sPESI score cannot be applied as a standalone rule to make that decision. A study has considered this difference between sPESI score and Hestia Criteria and demonstrated that 38% of patients were excluded from an outpatient treatment for subjective reasons, mainly due to comorbidities and social reasons, which may indicate the added value of integrating a subjective criterion when deciding for discharge of these patients. 13 Thus, besides HOME-PE's given evidence, a review that gathers all existing data about applying both strategies in the same patients may add value and provide a more insured decision-making for clinicians. This review fills this evidence gap by demonstrating that both standalone sPESI score and Hestia Criteria could be used to identify low-risk patients eligible for home treatment due to similar high negative predictive values with both strategies.

### Limitations to the Study

The first limitation regards the fact that this review only considered studies that applied both Hestia Criteria and sPESI score to the same population, limiting the comparison with other validated prognostic tools. This also led to the inclusion of a small number of studies and patients that were currently evaluated with both scores.

Second, despite the currently known importance of anticoagulation in the prognosis of PE, the data about anticoagulation was missing in two of the included studies. 9 11 The prediction of overall complications should also include data about anticoagulation and risk of bleeding, despite the absence of consensus of each risk stratification tool should be used in VTE patients. 14

All studies included patients with similar characteristics presenting with PE, except for one study that included patients with incidentally diagnosed PE. Even though those patients represent a small fraction of the study sample, this may introduce bias, as these patients would tend to be of lower risk.

Finally, this review is not powered to assess the real impact of early discharge since only one of these studies gave anticipated discharge for low-risk patients (and in this study, the decision for early discharge was not based on any of the criteria covered by this review). However, this review may offer a window to the expected outcomes of patients presenting with PE selected by these stratification rules to an early discharge or home treatment.

## Conclusion

Hestia Criteria and sPESI score have both a high sensitivity to adequately select low-risk pulmonary embolism patients for early discharge and/or home treatment. Even though the number of patients selected for this strategy by these scores may be inferior to the number of patients that would safely benefit from it, it contributes to safely reduce hospitalizations. The analysis of these results should take into account the small number of studies included in the review, and more comprehensive evidence is necessary to support the findings of this systematic review.
