# Supplementary material for: The Accuracy of Hestia and Simplified PESI to Predict the Prognosis in Pulmonary Embolism: Systematic Review with Meta-analysis
Source: TH Open. 2022 Oct 23;6(4):e347–53. doi: 10.1055/a-1942-2526 (PMC9593482; doi:10.1055/a-1942-2526)
Supplement: Supplementary file 1 — Supplementary Material [file 10-1055-a-1942-2526-s21100075.pdf]

Supplementary PRISMA

Topic	No.	Item	Location where item is reported
**TITLE**			
**Title**	1	Identify the report as a systematic review.	1
**ABSTRACT**			
**Abstract**	2	See the PRISMA 2020 for Abstracts checklist	
**INTRODUCTION**			
**Rationale**	3	Describe the rationale for the review in the context of existing knowledge.	3
**Objectives**	4	Provide an explicit statement of the objective(s) or question(s) the review addresses.	4
**METHODS**			
**Eligibility criteria**	5	Specify the inclusion and exclusion criteria for the review and how studies were grouped for the syntheses.	4
**Information sources**	6	Specify all databases, registers, Web sites, organisations, reference lists and other sources searched or consulted to identify studies. Specify the date when each source was last searched or consulted.	4,5
**Search strategy**	7	Present the full search strategies for all databases, registers and Web sites, including any filters and limits used.	5
**Selection process**	8	Specify the methods used to decide whether a study met the inclusion criteria of the review, including how many reviewers screened each record and each report retrieved, whether they worked independently, and if applicable, details of automation tools used in the process.	5
**Data collection process**	9	Specify the methods used to collect data from reports, including how many reviewers collected data from each report, whether they worked independently, any processes for obtaining or confirming data from study investigators, and if applicable, details of automation tools used in the process.	6
**Data items**	10a	List and define all outcomes for which data were sought. Specify whether all results that were compatible with each outcome domain in each study were sought (e.g., for all measures, time points, analyses), and if not, the methods used to decide which results to collect.	5
	10b	List and define all other variables for which data were sought (e.g., participant and intervention characteristics, funding sources). Describe any assumptions made about any missing or unclear information.	N/A
**Study risk of bias assessment**	11	Specify the methods used to assess risk of bias in the included studies, including details of the tool(s) used, how many reviewers assessed each study and whether they worked independently, and if applicable, details of automation tools used in the process.	6
**Effect measures**	12	Specify for each outcome the effect measure(s) (e.g., risk ratio, mean difference) used in the synthesis or presentation of results.	6
**Synthesis methods**	13a	Describe the processes used to decide which studies were eligible for each synthesis (e.g., tabulating the study intervention characteristics and comparing against the planned groups for each synthesis (item 5)).	6
	13b	Describe any methods required to prepare the data for presentation or synthesis, such as handling of missing summary statistics, or data conversions.	6
	13c	Describe any methods used to tabulate or visually display results of individual studies and syntheses.	6
	13d	Describe any methods used to synthesize results and provide a rationale for the choice(s). If meta-analysis was performed, describe the model(s), method(s) to identify the presence and extent of statistical heterogeneity, and software package(s) used.	6
	13e	Describe any methods used to explore possible causes of heterogeneity among study results (e.g., subgroup analysis, meta-regression).	N/A

 © 2022. The Author(s).

(*Continued*)

Topic	No.	Item	Location where item is reported
	13f	Describe any sensitivity analyses conducted to assess robustness of the synthesized results.	N/A
**Reporting bias assessment**	14	Describe any methods used to assess risk of bias due to missing results in a synthesis (arising from reporting biases).	N/A
**Certainty assessment**	15	Describe any methods used to assess certainty (or confidence) in the body of evidence for an outcome.	N/A
RESULTS			
**Study selection**	16a	Describe the results of the search and selection process, from the number of records identified in the search to the number of studies included in the review, ideally using a flow diagram.	5,7
	16b	Cite studies that might appear to meet the inclusion criteria, but which were excluded, and explain why they were excluded.	7
**Study characteristics**	17	Cite each included study and present its characteristics.	7
**Risk of bias in studies**	18	Present assessments of risk of bias for each included study.	8
**Results of individual studies**	19	For all outcomes, present, for each study: (a) summary statistics for each group (where appropriate) and (b) an effect estimate and its precision (e.g., confidence/credible interval), ideally using structured tables or plots.	8,9,10,11
**Results of syntheses**	20a	For each synthesis, briefly summarize the characteristics and risk of bias among contributing studies.	7,8
	20b	Present results of all statistical syntheses conducted. If meta-analysis was done, present for each the summary estimate and its precision (e.g., confidence/credible interval) and measures of statistical heterogeneity. If comparing groups, describe the direction of the effect.	8,9, supplementary
	20c	Present results of all investigations of possible causes of heterogeneity among study results.	N/A
	20d	Present results of all sensitivity analyses conducted to assess the robustness of the synthesized results.	N/A
**Reporting biases**	21	Present assessments of risk of bias due to missing results (arising from reporting biases) for each synthesis assessed.	N/A
**Certainty of evidence**	22	Present assessments of certainty (or confidence) in the body of evidence for each outcome assessed.	N/A
DISCUSSION			
**Discussion**	23a	Provide a general interpretation of the results in the context of other evidence.	12
	23b	Discuss any limitations of the evidence included in the review.	13
	23c	Discuss any limitations of the review processes used.	13
	23d	Discuss implications of the results for practice, policy, and future research.	13
OTHER INFORMATION			
**Registration and protocol**	24a	Provide registration information for the review, including register name and registration number, or state that the review was not registered.	N/A
	24b	Indicate where the review protocol can be accessed, or state that a protocol was not prepared.	N/A
	24c	Describe and explain any amendments to information provided at registration or in the protocol.	N/A
**Support**	25	Describe sources of financial or non-financial support for the review, and the role of the funders or sponsors in the review.	N/A
**Competing interests**	26	Declare any competing interests of review authors.	N/A
**Availability of data, code and other materials**	27	Report which of the following are publicly available and where they can be found: template data collection forms; data extracted from included studies; data used for all analyses; analytic code; any other materials used in the review.	N/A

 © 2022. The Author(s).

Supplementary Table S1 Studies' results

Study	Sample	30-day mortality	Stratification rule	Low-risk (%)	30-day mortality (%)	True Positive	False Negative	False Positive	True Negative	Sensitivity %	Scpecificity %	Positive predictive value %	Negative predictive value %	C-statistic
**Weeda et al**	**573**	33 (5.8%)	**sPESI**	177 (30.9)	1 (0.6)	32	1	364	176	97.0 (82.5–99.8)	32.6 (28.7–36.8)	8.1 (5–7-11.3)	99.4 (96.4–100)	0.731 (0.653–0.810)
			**Hestia**	160 (27.9)	0 (0.0)	33	0	380	160	100.0 (87.0–100)	29.6 (25.8–33.7)	8.0 (5.6–11.1)	100.0 (97.1–100)	0.791 (0.721–0.860)
**Vanni et al.**	**547**	44 (8.0%)	**sPESI**	100 (18.3)	1 (1.0) 2% recorre VTE 0% hemorragia major	43	1	404	99	97.7 (88.0–99.9)	19.7 (16.3–23.4)	9.6 (9.0–10.1)	99.0 (93.4–99.9)	–
			**Hestia**	228 (41.7)	5 (2.2) 0.4% recorre VTE 0% hemorragia major	39	5	280	223	88.6 (75.4–96.2)	44.3 (39.9–48.8)	12.2 (10.8–13.6)	97.8 (95.1–99.0)	–
**Quezada et al.**	**488**	31 (6.4%)	**sPESI**	135 (27.7)	1 (0.7)	30	1	323	134	96.8 (81.5–99.8)	29.3 (25.2–33.8)	8.5 (5.9–12.0)	99.3 (95.3–100)	0.63 (0.59–0.67)
			**Hestia**	132 (27.0)	3 (2.3)	28	3	328	129	90.3 (73.1–97.5)	28.2 (24.2–32.6)	7.9 (5.4–11.3)	97.7 (93.0–99.4)	0.59 (0.54–0.65)

Supplementary Table S2 Hestia's aggregate results

Parameter	Estimate	2.5% CI	97.5% CI
Sensitivity	0.923	0.843	0.964
Specificity	0.338	0.262	0.423
False Positive Rate	0.662	0.577	0.738
Diagnostic Odds Ratio	6.120	2.905	12.890
Likelihood Ratio +ve	1.394	1.244	1.562
Likelihood Ratio -ve	0.228	0.115	0.453

Supplementary Table S3 sPESI's aggregate results

Parameter	Estimate	2.5% CI	97.5% CI
Sensitivity	0.972	0.917	0.991
Specificity	0.269	0.209	0.338
False Positive Rate	0.731	0.662	0.791
Diagnostic Odds Ratio	12.860	3.898	42.429
Likelihood Ratio +ve	1.329	1.211	1.460
Likelihood Ratio -ve	0.103	0.033	0.324

Supplementary Table S4 Inconsistency of pooled results

Inconsistency [I^2; %]	Hestia	sPESI
Sensitivity	94.2%	91.7%
Specificity	67.6%	0.0%
Positive LR	0.0%	0.0%
Negative LR	69.1%	82.3%

Abbreviations: LR, Likelihood ratio; sPESI, simplified Pulmonary Embolism Severity Index.

 © 2022. The Author(s).

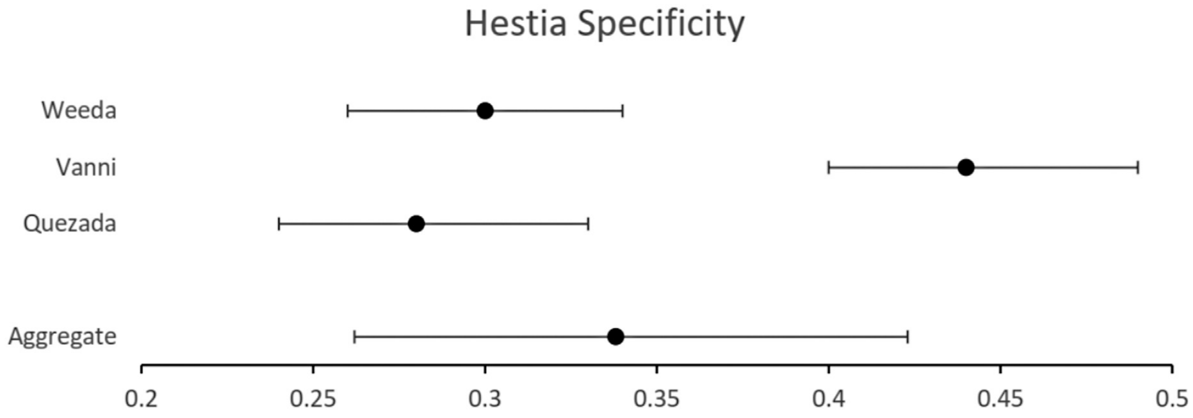

Supplementary Fig. S1: Hestia specificity.

Supplementary Fig. S1 Hestia specificity.

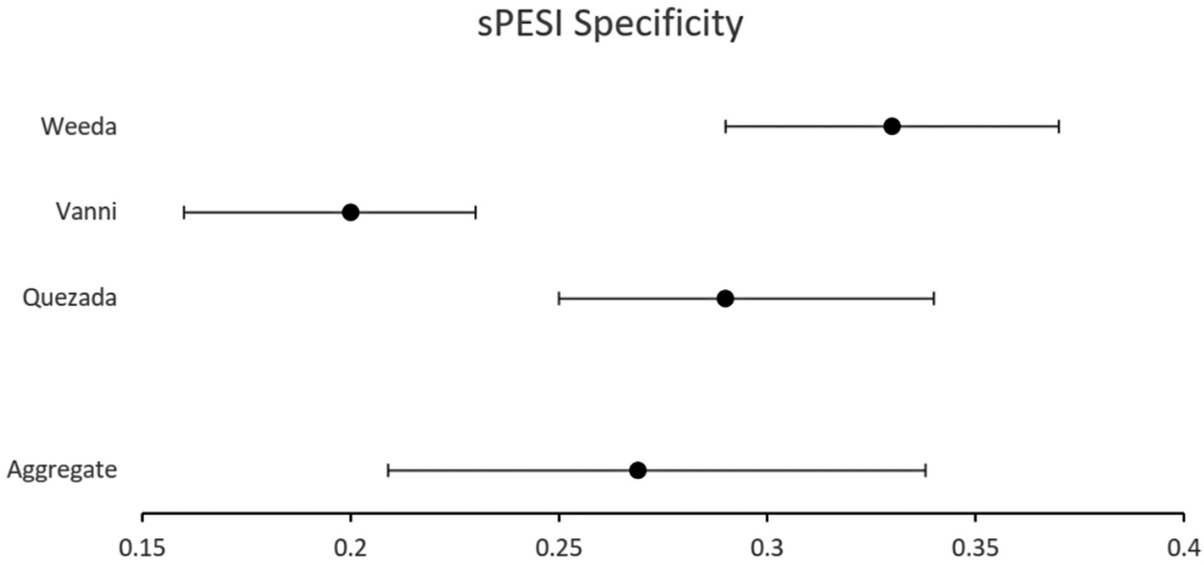

Supplementary Fig. S2 sPESI specificity.

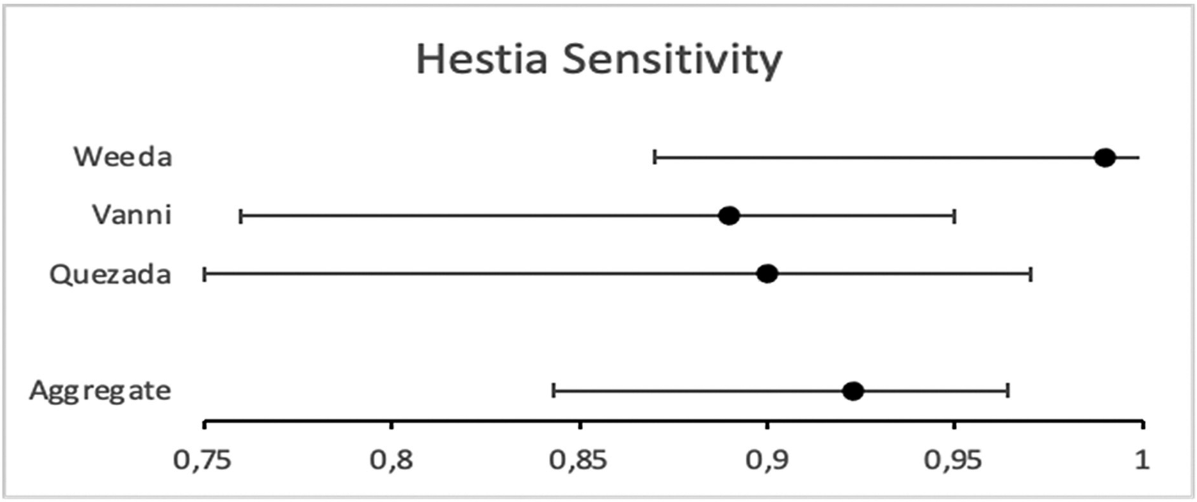

Supplementary Fig. S3 Hestia sensitivity.

 © 2022. The Author(s).

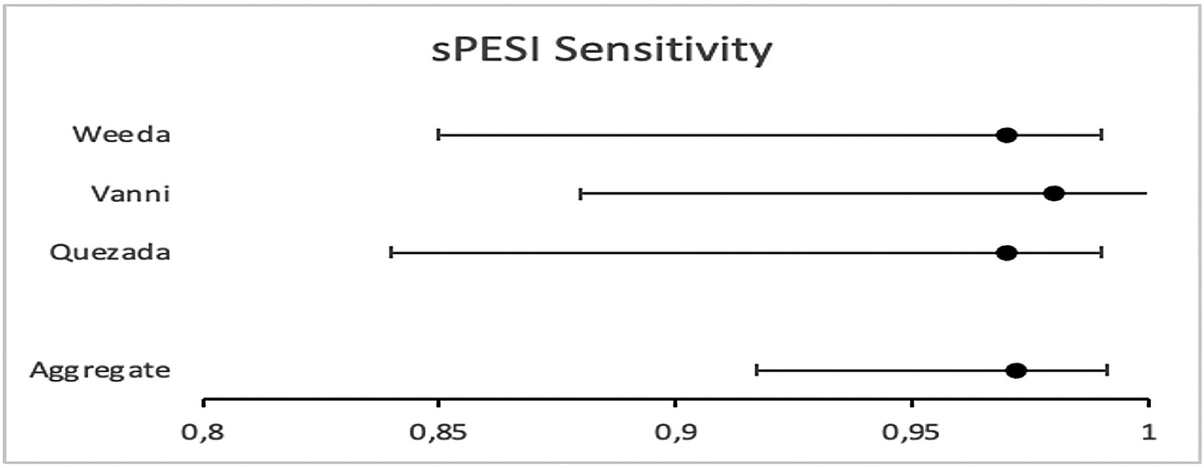

Supplementary Fig. S4 sPESI sensitivity.

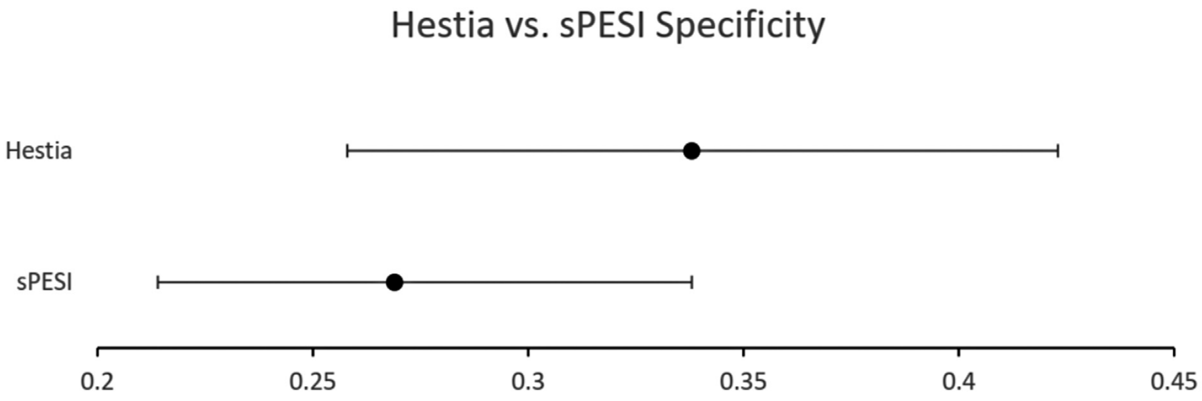

Supplementary Fig. S5 Hestia versus sPESI specificity.

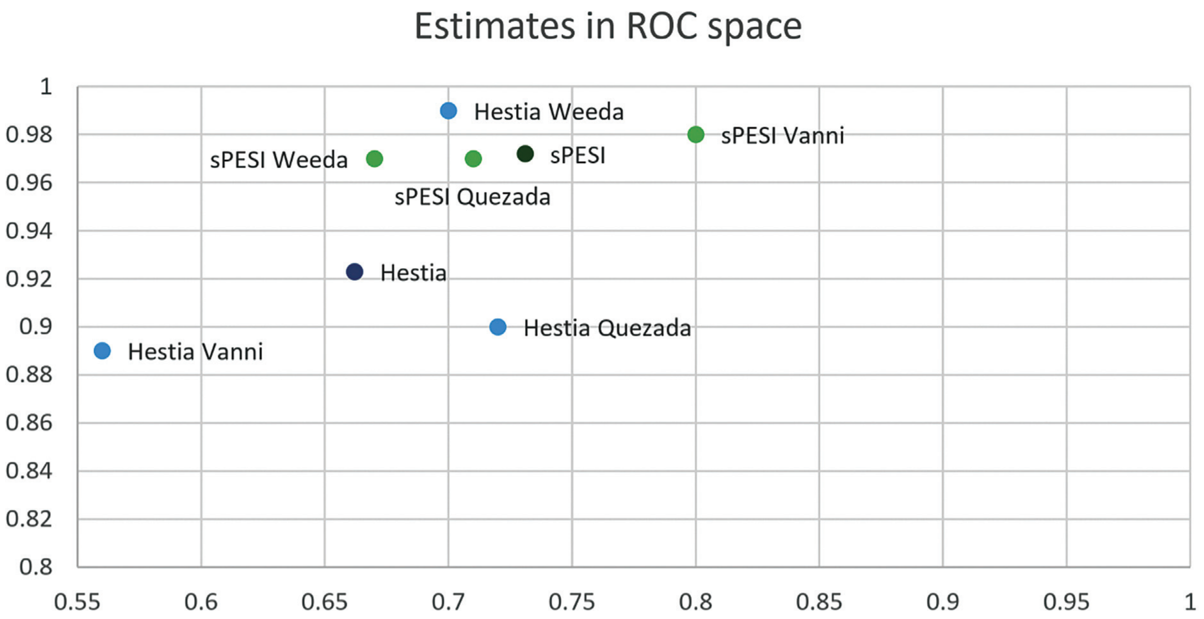

Supplementary Fig. S6 Hestia and sPESI estimates in receiver operating characteristic plane.

 © 2022. The Author(s).

QUADAS-2 (Weeda et al.)

Phase 1: State the review question:

Patients (setting, intended use of index test, presentation, prior testing): Consecutive patients at least 18 years of age admitted to the hospital with a primary diagnosis of acute pulmonary embolism. There were excluded subjects submitted to thrombolysis and/or pulmonary embolectomy within the first 48h after the acute event.<br>The index tests aim to identify patients who are at low risk for early mortality, to consider early discharge and provide an anticoagulation strategy in an outpatient setting.<br>All patients were managed according to usual clinical practice for that institution (Hartford Hospital, Connecticut, USA).<br>The index tests were applied a posteriori, using information collected as close as possible to the index pulmonary embolism.<br>For the diagnosis of the pulmonary embolism were used perfusion-ventilation lung scan, computed tomography pulmonary angiography or pulmonary angiography. On the event of the previous testing were non-diagnostic, compression ultrasonography of the ower extremities was performed.
Index test(s): Hestia criteria, simplified Pulmonary Embolism Severity Index (sPESI), Pulmonary Embolism Severity Index, In-hospital Mortality for Pulmonary Embolism using Claims Data (IMPACT)
Reference standard and target condition: The reference standard for mortality within the first 30 days of pulmonary embolism presentation is mortality itself.

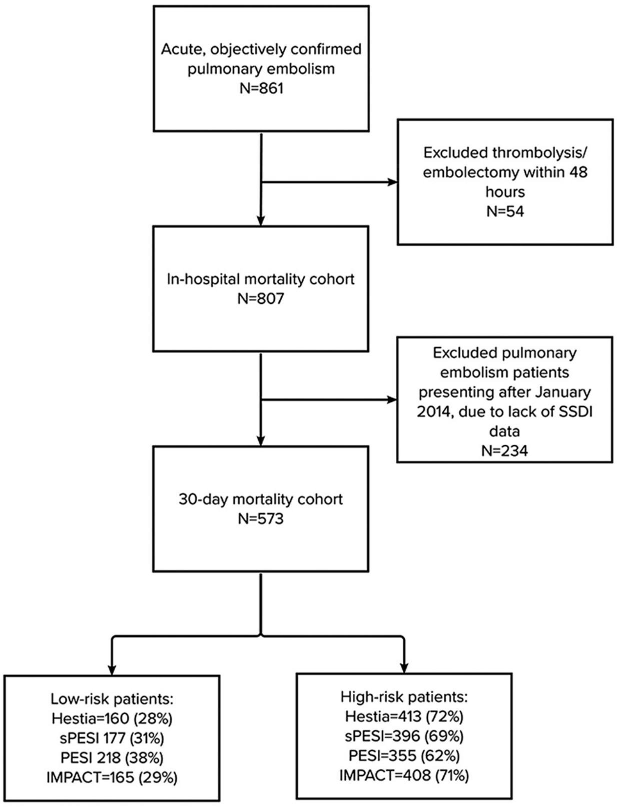

Phase 2: Draw a flow diagram for the primary study

 © 2022. The Author(s).

Phase 3: Risk of bias and applicability judgements

QUADAS-2 is structured so that 4 key domains are each rated in terms of risk of bias and the concern regarding applicability to the research question (as defined above). Each key domain has a set of signaling questions to help reach the judgments regarding bias and applicability.

Domain 1: Patient selection	
**A. Risk of Bias**	
Describe methods of patient selection: There were included consecutive patients at least 18 years of age admitted to the hospital with a primary diagnosis of acute pulmonary embolism. There were excluded subjects submitted to thrombolysis and/or pulmonary embolectomy within the first 48h after the acute event, since this put them in a high risk category according to current guidelines.<br>From the 30-day mortality cohort were excluded patients admitted posteriorly to January 2014 on the impossibility of accessing mortality data from the Social Security Death Index platform, due to changes in rules regarding access to data.	
Was a consecutive or random sample of patients enrolled?	Yes
Was a case-control design avoided?	Yes
Did the study avoid innapropriate exclusions?	Yes
Could the selection of patients have introduced bias?	Risk: Low
**B. Concers regarding applicability**	
Describe included patients (prior testing, presentation, intended use of index test and setting): Consecutive patients at least 18 years of age admitted to the hospital with a primary diagnosis of acute pulmonary embolism. There were excluded subjects submitted to thrombolysis and/or pulmonary embolectomy within the first 48h after the acute event.<br>The index tests aim to identify patients who are at low risk for early mortality, to consider early discharge and provide an anticoagulation strategy in an outpatient setting.<br>All patients were managed according to usual clinical practice for that institution (Hartford Hospital, Connecticut, USA).<br>The index tests were applied a posteriori, using information collected as close as possible to the index pulmonary embolism.<br>For the diagnosis of the pulmonary embolism were used perfusion-ventilation lung scan, computed tomography pulmonary angiography or pulmonary angiography. On the event of the previous testing were non-diagnostic, compression ultrasonography of the ower extremities was performed.	
Is there concern that the included patients do not match the review question?	Concern: Low

Domain 2: Index Test - Hestia Criteria	
**A. Risk of Bias**	
Describe the index test and how it was conducted and interpreted: The Hestia Criteria adresses clinical factors that may interfere with early-mortality. It includes 11 items regarding haemodynamically instability, necessity of thrombolysis or embolectomy, high risk for bleeding, need for oxygen supply to maintain oxygen saturation >90% >24 hour, pulmonary embolism diagnosed during anticoagulant treatment, intravenous pain medication >24 hour, medical or social reason for treatment in the hospital >24 hour, creatinine clearance of less than 30 ml/min, severe liver impairment, pregnancy, and documented history of heparin-induced thrombocytopenia. If one of these items is fulfilled the patient cannot be classified into the low-risk category.<br>There are some items that are, to some point subjective, and may be assessed differently by different clinicians.<br>All data was retrieved from the electronic health record by trained study personnel blinded to the study outcome.<br>Index test cut-offs were determined *a priori* and according to the test certification studies.	
Were the index test results interpreted without knowledge of the results of the reference standard?	Yes
If a threshold was used, was it pre-specified?	Yes
Could the conduct or interpretation of the index test have introduced bias?	Risk: Low
**B. Concerns regarding applicability**	
Is there concern that the index, its conduct, or interpretation differ from the review question?	Concern: Low

 © 2022. The Author(s).

Domain 2: Index Test - sPESI<br>If more than one index test was used, please complete for each test.	
**A. Risk of Bias**	
Describe the index test and how it was conducted and interpreted: sPESI is a clinical stratification rule that takes into account some particular variables included in the PESI score. It attributes one point to the presence of each of the following variables: age above 80 years old, history of cancer, history of chronic cardiopulmonary disease, pulse >= 110 beats/min, systolic blood pressure <100mmHg, arterial oxyhemoglobin saturation level <90%. Patients were included in the low-risk category if none of these factors were present.<br>All data was retrieved from the electronic health record by trained study personnel blinded to the study outcome.<br>Index test cut-offs were determined *a priori* and according to the test certification studies.	
Were the index test results interpreted without knowldege of the results of the reference standard?	Yes
If a threshold was used, was it pre-specified?	Yes/Unclear
Could the conduct or interpretation of the index test have introduced bias?	Risk: Low
**B. Concerns regarding applicability**	
Is there concern that the index, its conduct, or interpretation differ from the review question?	Concern: Low

Domain 2: Index Test - PESI<br>If more than one index test was used, please complete for each test.	
**A. Risk of Bias**	
Describe the index test and how it was conducted and interpreted: PESI is a clinical stratification rule in which each risk variable is assigned a classification. Age above 80 years (one point for each year above), male sex (10 points), history of cancer (30 points), history of heart failure (10 points), history of chorinc lung disese (10 points), Pulse >= 110 beats/min (20 points), systolic blood pressure <100mmHg (30 points), respiratory rate >= 30 breaths/min (20 points), temperature <36°C (20 points), altered mental status (60 points), arterial oxyhemoglobin saturation level <90% (20 points). The added points result in a score. For this particular study, patients were defined as low-risk if this score was below 85 points.<br>All data was retrieved from the electronic health record by trained study personnel blinded to the study outcome.<br>Index test cut-offs were determined *a priori* and according to the test certification studies.	
Were the index test results interpreted without knowldege of the results of the reference standard?	Yes
If a threshold was used, was it pre-specified?	Yes/Unclear
Could the conduct or interpretation of the index test have introduced bias?	Risk: Low
**B. Concerns regarding applicability**	
Is there concern that the index, its conduct, or interpretation differ from the review question?	Concern: Low

Domain 2: Index Test - IMPACT<br>If more than one index test was used, please complete for each test.	
**A. Risk of Bias**	
Describe the index test and how it was conducted and interpreted: IMPACT is a stratification tool that is based on patients history and comorbidities to attribute a risk of early mortality in pulmonary embolism. This risk is estimated through a formula in which estimated % absolute risk = 1/(1 + exp(-x); where x = – 5.833 + (0.026*age) + (0.402*myocardial infarction) + (0.368*chronic lung disease) + (0.464*stroke) + (0.6 38*prior major bleeding) + (0.298*atrial fibrillation) + (1.06 1*cognitive impairment) + (0.554*heart failure) + (0.364* renal failure) + (0.484*liver disease) + (0.523*coagulopa- thy) + (1.068*cancer).<br>A 30 day-mortality calculated risk below 1.5% was admitted to the low-risk category.<br>All data was retrieved from the electronic health record by trained study personnel blinded to the study outcome.<br>Index test cut-offs were determined *a priori*	
Were the index test results interpreted without knowldege of the results of the reference standard?	Yes
If a threshold was used, was it pre-specified?	Yes/Unclear
Could the conduct or interpretation of the index test have introduced bias?	Risk: Low
**B. Concerns regarding applicability**	
Is there concern that the index, its conduct, or interpretation differ from the review question?	Concern: Low

 © 2022. The Author(s).

Domain 3: Reference Standard	
**A. Risk of Bias**	
Describe the reference standard and how it was conducted and interpreted: The reference standard is mortality. Information on patients mortality within the first 30 days after the acute event was retrieved from the Social Security Death Index.	
Is the reference standard likely to correctly classify the target condition?	Yes
Were the reference standard results interpreted without knowledge of the results of the index test?	Yes
Could the reference standard, its conduct, or interpretation have introduce bias?	Risk: Low
**B. Concerns regarding applicability**	
Is there concern that the target condition as defined by the reference standard does not match the review question?	Concern: Low

Domain 4: Flow and Timing	
**A. Risk of Bias**	
Describe any patients who did not receive the index test(s) and/or reference standard or who were excluded from the 2 × 2 table (refer to flow diagram): From the patients included in the study, none was excluded from the index tests or mortality analysis.	
Describe the time interval and any interventions between index test(s) and reference standard: Patients were managed through standard-of-care of that institution. Reference standard is assessed 30 days after the acute event, while the index tests were applied using information as close as possible to the pulmonary embolism initial presentation.<br>Since this was a retrospective study, the testing performed had no risk of bias.	
Was there an appropriate interval between index test(s) and reference standard?	Yes
Did all patients receive a reference standard?	Yes
Did patients receive the same reference standard?	Yes
Were all patients included in the analysis?	Yes
Could the patient flow have introduced bias?	Risk: Low

 © 2022. The Author(s).

QUADAS-2 (Vanni et al.)

Phase 1: State the review question:

Patients (setting, intended use of index test, presentation, prior testing): Consecutive adult patients with objectively confirmed pulmonary embolism (included those with incidental discovery of the condition)<br>The index tests aim to identify patients who are at low risk for early mortality, to consider early discharge and provide an anticoagulation strategy in an outpatient setting.<br>The management of patients and decision regarding early discharge was in charge of the attending physician. The four centers of care didn't routinely use clinical stratification tools such as Hestia criteria, simplified Pulmonary Embolism Severity Index (sPESI) or Pulmonary Embolism Severity Index (PESI) for decision making.<br>Hestia Criteria, sPESI and PESI were assessed by an investigator after patient inclusion in the study.<br>Patients were also submitted to echocardiography or computed tomography pulmonary angiography to evaluate heart function (more particularly, to assess right ventricular dysfunction, an assumed prognostic factor). Some patients were submited to cardiac troponin testing.
Index test(s): Hestia criteria, simplified Pulmonary Embolism Severity Index (sPESI), Clinical gestalt
Reference standard and target condition: The reference standard for early complications of pulmonary embolism are venous thromboembolism recurrence, major bleeding and all-cause death within 30 days.

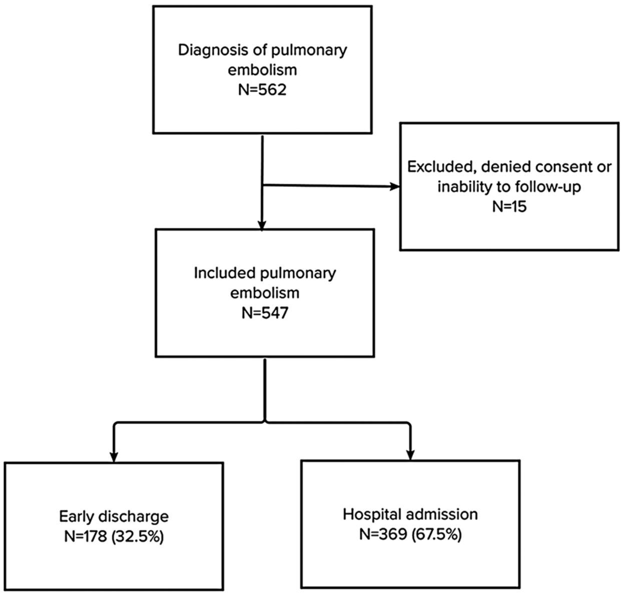

Phase 2: Draw a flow diagram for the primary study

Phase 3: Risk of bias and applicability judgements

Domain 1: Patient selection	
**A. Risk of Bias**	
Describe methods of patient selection: There were included consecutive adult patients with objectively confirmed pulmonary embolism. Subjects incidentally diagnosed with pulmonary embolism were also included in the study	
Was a consecutive or random sample of patients enrolled?	Yes
Was a case-control design avoided?	Yes
Did the study avoid innapropriate exclusions?	Yes
Could the selection of patients have introduced bias?	Risk: Low
**B. Concers regarding applicability**	
Patients (setting, intended use of index test, presentation, prior testing): Consecutive adult patients with objectively confirmed pulmonary embolism (included those with incidental discovery of the condition)<br>The index tests aim to identify patients who are at low risk for early mortality, to consider early discharge and provide an anticoagulation strategy in an outpatient setting.<br>The management of patients and decision regarding early discharge was in charge of the attending physician. The four centers of care didn't routinely use clinical stratification tools such as Hestia criteria, simplified Pulmonary Embolism Severity Index (sPESI) or Pulmonary Embolism Severity Index (PESI) for decision making.<br>Hestia Criteria, sPESI and PESI were assessed by an investigator after patient inclusion in the study.	
Is there concern that the included patients do not match the review question?	Concern: Low

 © 2022. The Author(s).

Domain 2: Index Test - Hestia Criteria	
**A. Risk of Bias**	
Describe the index test and how it was conducted and interpreted: The Hestia Criteria adresses clinical factors that may interfere with early-mortality. It includes 11 items regarding haemodynamically instability, necessity of thrombolysis or embolectomy, high risk for bleeding, need for oxygen supply to maintain oxygen saturation >90% >24 hour, pulmonary embolism diagnosed during anticoagulant treatment, intravenous pain medication >24 hour, medical or social reason for treatment in the hospital >24 hour, creatinine clearance of less than 30 ml/min, severe liver impairment, pregnancy, and documented history of heparin-induced thrombocytopenia. If one of these items is fulfilled the patient cannot be classified into the low-risk category.<br>There are some items that are, to some point subjective, and may be assessed differently by different clinicians.<br>All data was retrieved from the electronic health record by trained study personnel blinded to the study outcome.<br>Index test cut-offs were determined *a priori* and according to the test certification studies.	
Were the index test results interpreted without knowledge of the results of the reference standard?	Yes
If a threshold was used, was it pre-specified?	Yes
Could the conduct or interpretation of the index test have introduced bias?	Risk: Low
**B. Concerns regarding applicability**	
Is there concern that the index, its conduct, or interpretation differ from the review question?	Concern: Low

Domain 2: Index Test - sPESI<br>If more than one index test was used, please complete for each test.	
**A. Risk of Bias**	
Describe the index test and how it was conducted and interpreted: sPESI is a clinical stratification rule that takes into account some particular variables included in the PESI score. It attributes one point to the presence of each of the following variables: age above 80 years old, history of cancer, history of chronic cardiopulmonary disease, pulse >= 110 beats/min, systolic blood pressure <100mmHg, arterial oxyhemoglobin saturation level <90%. Patients were included in the low-risk category if none of these factors were present.<br>All data was retrieved from the electronic health record by trained study personnel blinded to the study outcome.<br>Index test cut-offs were determined *a priori* and according to the test certification studies.	
Were the index test results interpreted without knowledge of the results of the reference standard?	Yes
If a threshold was used, was it pre-specified?	Yes
Could the conduct or interpretation of the index test have introduced bias?	Risk: Low
**B. Concerns regarding applicability**	
Is there concern that the index, its conduct, or interpretation differ from the review question?	Concern: Low

Domain 2: Index Test - PESI<br>If more than one index test was used, please complete for each test.	
**A. Risk of Bias**	
Describe the index test and how it was conducted and interpreted: PESI is a clinical stratification rule in which each risk variable is assigned a classification. Age above 80 years (one point for each year above), male sex (10 points), history of cancer (30 points), history of heart failure (10 points), history of chorinc lung disese (10 points), Pulse >= 110 beats/min (20 points), systolic blood pressure <100mmHg (30 points), respiratory rate >= 30 breaths/min (20 points), temperature <36°C (20 points), altered mental status (60 points), arterial oxyhemoglobin saturation level <90% (20 points). The added points result in a score. For this particular study, patients were defined as low-risk if this score was below 85 points.<br>All data was retrieved from the electronic health record by trained study personnel blinded to the study outcome.<br>Index test cut-offs were determined *a priori* and according to the test certification studies.	
Were the index test results interpreted without knowledge of the results of the reference standard?	Yes
If a threshold was used, was it pre-specified?	Yes/Unclear
Could the conduct or interpretation of the index test have introduced bias?	Risk: Low
**B. Concerns regarding applicability**	
Is there concern that the index, its conduct, or interpretation differ from the review question?	Concern: Low

 © 2022. The Author(s).

Domain 2: Index Test - Clinical Gestalt<br>If more than one index test was used, please complete for each test.	
**A. Risk of Bias**	
Describe the index test and how it was conducted and interpreted: This study compared clinical classification tools with clinical gestalt. The attending physician would be in charge of the management of the patients, and whether he would have early discharge to continue anticoagulation treatment in an outpatient setting. He made this decision based on patient history, clinical evaluation, the results of general blood tests, evaluation of right ventricular function, as patient's anticipated compliance.	
Were the index test results interpreted without knowledge of the results of the reference standard?	Yes
If a threshold was used, was it pre-specified?	There isn't a stabilified scoring system that allowed patient evaluation and stratification
Could the conduct or interpretation of the index test have introduced bias?	Risk: Yes
**B. Concerns regarding applicability**	
Is there concern that the index, its conduct, or interpretation differ from the review question?	Concern: Low

Domain 3: Reference Standard	
**A. Risk of Bias**	
Describe the reference standard and how it was conducted and interpreted: The reference standard for early complications of pulmonary embolism are venous thromboembolism recurrence, major bleeding and all-cause death within 30 days.<br>Recurrent pulmonary embolism was determined as the presence of a new intraluminal filling defect on computed tomographic angiography, or by a new perfusion scan defect involving >75% of a lung segment on a ventilation-perfusion scan.<br>Recurrent deep vein thrombosis was determined as a new intraluminal fiilling defect on compression ultrasonography of symptomatic upper or lower limbs.<br>Major bleeding was defined according to the Internal Society of Thrombosis and Haemosthasis definition: acute clinically overt bleeding associated with one or more of the following: decrease in hemoglobin of 2 g/dL or more; transfusion of 2 or more units of packed red blood cells; bleeding that occurs ins a critical site (instracranial/intra-spinal, intraocular, pericardial, intra-articular, intramuscular with compartment syndrome, or retroperitoneal); bleeding that is fatal, or bleeding that necessitates acute surgical intervention.<br>All suspected adverse events and deaths were evaluated by a local adjudication committee.	
Is the reference standard likely to correctly classify the target condition?	Yes
Were the reference standard results interpreted without knowledge of the results of the index test?	Yes
Could the reference standard, its conduct, or interpretation have introduce bias?	Risk: Low
**B. Concerns regarding applicability**	
Is there concern that the target condition as defined by the reference standard does not match the review question?	Concern: Low

Domain 4: Flow and Timing	
**A. Risk of Bias**	
Describe any patients who did not receive the index test(s) and/or reference standard or who were excluded from the 2 × 2 table (refer to flow diagram): From the patients who met criteria to be included in the study, 10 were excluded because they denied consent, and 5 were excluded by inability to be followed up.	
Describe the time interval and any interventions between index test(s) and reference standard: Index tests were evaluated by a local investigator following the patient admission in te study. It did not interfere with the decision to early discharge and was performed before the appearance of adverse events or death (the reference standard)	
Was there an appropriate interval between index test(s) and reference standard?	Yes
Did all patients receive a reference standard?	Yes
Did patients receive the same reference standard?	Yes
Were all patients included in the analysis?	Yes
Could the patient flow have introduced bias?	Risk: Low

 © 2022. The Author(s).

QUADAS-2 (Quezada et al.)

Phase 1: State the review question:

Patients (setting, intended use of index test, presentation, prior testing): Patients presenting with acute symptomatic pulmonary embolism confirmed by objective testing admitted to the emergency department.<br>For the diagnosis of the pulmonary embolism were used ventilation-perfusion lung scan, or computed tomography pulmonary angiography.<br>On the event of the previous testing were non-diagnostic, compression ultrasonography of the lower extremities was performed.<br>The index tests aim to identify patients who are at low risk for early mortality, to consider early discharge and provide an anticoagulation strategy in an outpatient setting.
Index test(s): Hestia criteria, simplified Pulmonary Embolism Severity Index (sPESI)
Reference standard and target condition: The reference standard for mortality within the first 30 days of pulmonary embolism presentation is mortality itself.

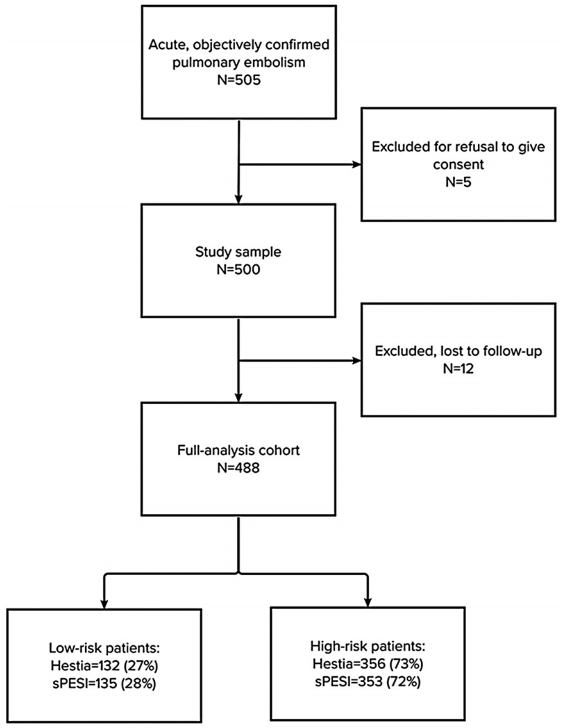

Phase 2: Draw a flow diagram for the primary study

Phase 3: Risk of bias and applicability judgements

Domain 1: Patient selection	
**A. Risk of Bias**	
Describe methods of patient selection: There were included consecutive patients admitted to the emergency department hospital with symptomatic acute pulmonary embolism.	
Was a consecutive or random sample of patients enrolled?	Yes
Was a case-control design avoided?	Yes
Did the study avoid innapropriate exclusions?	Yes
Could the selection of patients have introduced bias?	Risk: Low
**B. Concers regarding applicability**	
Describe included patients (prior testing, presentation, intended use of index test and setting): Patients presenting with acute symptomatic pulmonary embolism confirmed by objective testing admitted to the emergency department.<br>For the diagnosis of the pulmonary embolism were used ventilation-perfusion lung scan, or computed tomography pulmonary angiography.<br>On the event of the previous testing were non-diagnostic, compression ultrasonography of the lower extremities was performed.<br>The index tests aim to identify patients who are at low risk for early mortality, to consider early discharge and provide an anticoagulation strategy in an outpatient setting.	
Is there concern that the included patients do not match the review question?	Concern: Low

 © 2022. The Author(s).

Domain 2: Index Test - Hestia Criteria	
**A. Risk of Bias**	
Describe the index test and how it was conducted and interpreted: The Hestia Criteria adresses clinical factors that may interfere with early-mortality. It includes 11 items regarding haemodynamically instability, necessity of thrombolysis or embolectomy, high risk for bleeding, need for oxygen supply to maintain oxygen saturation >90% >24 hour, pulmonary embolism diagnosed during anticoagulant treatment, intravenous pain medication >24 hour, medical or social reason for treatment in the hospital >24 hour, creatinine clearance of less than 30 ml/min, severe liver impairment, pregnancy, and documented history of heparin-induced thrombocytopenia. If one of these items is fulfilled the patient cannot be classified into the low-risk category. There are some items that are, to some point subjective, and may be assessed differently by different clinicians. Index test cut-offs were determined *a priori* and according to the test certification studies.	
Were the index test results interpreted without knowledge of the results of the reference standard?	Yes
If a threshold was used, was it pre-specified?	Yes
Could the conduct or interpretation of the index test have introduced bias?	Risk: Low
**B. Concerns regarding applicability**	
Is there concern that the index, its conduct, or interpretation differ from the review question?	Concern: Low

Domain 2: Index Test - sPESI<br>If more than one index test was used, please complete for each test.	
**A. Risk of Bias**	
Describe the index test and how it was conducted and interpreted: sPESI is a clinical stratification rule that takes into account some particular variables included in the PESI score. It attributes one point to the presence of each of the following variables: age above 80 years old, history of cancer, history of chronic cardiopulmonary disease, pulse >= 110 beats/min, systolic blood pressure <100mmHg, arterial oxyhemoglobin saturation level <90%. Patients were included in the low-risk category if none of these factors were present. Index test cut-offs were determined *a priori* and according to the test certification studies.	
Were the index test results interpreted without knowldege of the results of the reference standard?	Yes
If a threshold was used, was it pre-specified?	Yes/Unclear
Could the conduct or interpretation of the index test have introduced bias?	Risk: Low
**B. Concerns regarding applicability**	
Is there concern that the index, its conduct, or interpretation differ from the review question?	Concern: Low

Domain 3: Reference Standard	
**A. Risk of Bias**	
Describe the reference standard and how it was conducted and interpreted: The reference standard is mortality in the period 30 days after the onset of acute pulmonary embolism. This information was retrieved using patient or proxy interviews, and/or hospital chart review.	
Is the reference standard likely to correctly classify the target condition?	Yes
Were the reference standard results interpreted without knowledge of the results of the index test?	Yes
Could the reference standard, its conduct, or interpretation have introduce bias?	Risk: Low
**B. Concerns regarding applicability**	
Is there concern that the target condition as defined by the reference standard does not match the review question?	Concern: Low

 © 2022. The Author(s).

Domain 4: Flow and Timing	
**A. Risk of Bias**	
Describe any patients who did not receive the index test(s) and/or reference standard or who were excluded from the 2 × 2 table (refer to flow diagram): From the patients included in the study, none was excluded from the index tests or mortality analysis.	
Describe the time interval and any interventions between index test(s) and reference standard: Mortality, the reference standard, is assessed 30 days after the acute event, while the index tests were applied using information as close as possible to the pulmonary embolism initial presentation.	
Was there an appropriate interval between index test(s) and reference standard?	Yes
Did all patients receive a reference standard?	Yes
Did patients receive the same reference standard?	Yes
Were all patients included in the analysis?	Yes
Could the patient flow have introduced bias?	Risk: Low

Supplementary File: Search Strategy

#	**Searches**
1	Hestia.af.
2	spesi.af.
3	simplified pesi.af.
4	1 or 2 or 3
5	remove duplicates from 4

No language or timeline restriction.

 © 2022. The Author(s).
